# Development of marker‐free rice with stable and high resistance to rice black‐streaked dwarf virus disease through RNA interference

**DOI:** 10.1111/pbi.13459

**Published:** 2020-08-17

**Authors:** Zhiming Feng, Meng Yuan, Jie Zou, Lin‐Bo Wu, Lang Wei, Taiyu Chen, Nana Zhou, Wenxia Xue, Yafang Zhang, Zongxiang Chen, Keming Hu, Guo‐Liang Wang, Wende Liu, Xuebiao Pan, Shimin Zuo

**Affiliations:** ^1^ Key Laboratory of Plant Functional Genomics of the Ministry of Education/Jiangsu Key Laboratory of Crop Genomics and Molecular Breeding Agricultural College of Yangzhou University Yangzhou China; ^2^ Co‐Innovation Center for Modern Production Technology of Grain Crops of Jiangsu Province/Key Laboratory of Crop Genetics and Physiology of Jiangsu Province Yangzhou University Yangzhou China; ^3^ National Key Laboratory of Crop Genetic Improvement and National Center of Plant Gene Research College of Life Science and Technology Huazhong Agricultural University Wuhan China; ^4^ Institute for Molecular Physiology Heinrich Heine University of Düsseldorf Düsseldorf Germany; ^5^ Department of Plant Pathology Ohio State University Columbus Ohio USA; ^6^ State Key Laboratory for biology of plant diseases and insect pests/Institute of plant protection Chinese academy of agricultural sciences Beijing China; ^7^ Joint International Research Laboratory of Agriculture and Agri‐Product Safety the Ministry of Education of China Institutes of Agricultural Science and Technology Development Yangzhou University Yangzhou China

**Keywords:** *Oryza sativa* L, rice black‐streaked dwarf virus disease, RNA interference, marker‐free transgenic rice, high resistance

## Abstract

The rice black‐streaked dwarf virus (RBSDV) disease causes severe rice yield losses in Asia. RNA interference (RNAi) has been widely applied to develop antiviral varieties in plants. So far, only a few studies reported the application of RNAi in rice against RBSDV and most of them are lack of enough data to support its breeding potential, which limited the progress on developing RBSDV‐resistant variety. In this study, we generated three RNAi constructs to specifically target three RBSDV genes (S1, S2 and S6), respectively. We confirmed that RNAi targeting RBSDV S6 conferred rice with almost full immunity to RBSDV through phenotyping test in eight consecutive years in both artificial inoculation and field trials, while RNAi of S1 or S2 only leads to partially increased resistance. The S6RNAi was also found conferring strong resistance to southern rice black‐streaked dwarf virus (SRBSDV), a novel species closely related to RBSDV that outbroke recently in Southern China. In particular, no adverse effects on agronomical and developmental traits were found in S6RNAi transgenic lines. The marker‐free transgenic lines with S6RNAi, driven by either maize ubiquitin‐1 promoter or rice rbcS green tissue expression promoter, in elite rice background should have great potential in breeding of resistant varieties to both RBSDV and SRBSDV and provide a basis for further safety evaluation and commercial application.

Dear Editor,

Rice black‐streaked dwarf virus (RBSDV) disease transmitted by small brown planthoppers (*Laodelphax striatellus* Fallén, SBPH), causes severe rice yield losses in Asia (Zhou *et al*., 2015). Breeding resistant cultivars are one of the most economical and effective strategies to control the disease. In the past two decades, there were several studies on the identification of cultivars and the detection of QTLs for RBSDV resistance (Feng *et al*., 2019). However, few highly resistant germplasms or genes have been found (Sun *et al*., 2017), severely hindering the development of elite varieties with high RBSDV resistance through either conventional breeding or marker‐assisted selection (MAS) breeding.

RNA interference (RNAi), an evolutionarily conserved defence mechanism against RNA viruses, has been successfully applied to develop antiviral varieties in plants (Cristina *et al*., 2018). The genome of RBSDV contains 10 double‐stranded RNA (dsRNA) segments designated *S1*–*S10* (Wang *et al*., 2003). Previously, Shimizu *et al*. (2011a) generated RBSDV‐resistant transgenic rice by silencing *S9‐1*. Wang *et al*. (2016) constructed an RNAi vector simultaneously targeting 4 viral genes (*S1*, *S2*, *S6* and *S10*) and obtained high RBSDV‐resistant rice lines. However, it is not clear that targeting which specific one or the combination of four genes leading to the high resistance. Among the RNAi studies against the virus, not all RNAi constructs targeting any of the virus genes were equally effective (Shimizu *et al*., 2011b). Therefore, it is crucial to identify the right targets and design effective RNAi fragments for a specific viral gene.

For more than 10 years, our group has been committed to developing varieties with high RBSDV resistance. We generated three RNAi constructs targeting the specific fragments of RBSDV on *S1*, *S2* and *S6*, respectively, and transferred them into the highly susceptible *japonica* variety Wulingjing 1 (WLJ1). Three independent transgenic lines for each target with the accumulation of expected small interference RNA (siRNA) were selected for phenotyping test (Figure 1a). We first conducted an artificial inoculation using SBPH carrying an RBSDV viruliferous rate (VR) of 32% in 2010, then a large‐scale field trial in 2011 in Yangzhou with a VR of 9.4%. We found that RNAi of *S1* and *S2* individually had partial effects, whereas RNAi of *S6* conferred nearly full immunity to RBSDV (Figure 1b‐c; China patent: ZL201310202564.9). To further evaluate the stability of the *S6RNAi* lines, we conducted field trials from 2012 to 2013 at 4 different locations randomly selected in Jiangsu and Shandong provinces where the RBSDV disease had severe outbreaks since 2009. We observed that the *S6RNAi* lines showed stable resistance to RBSDV in all 4 locations (Figure 1d and e), indicating its potential for practical application. In a further field trial in 2014 at Kaifeng, Henan province, which had a VR of 12%, the *S6RNAi* lines with a diseased/total plant ratio (DPR) of less than 0.3%, displayed drastically higher resistance than a commercialized *japonica* rice variety Liangjing 6 (LG6) with a DPR of 36.7%, which is considered as a relatively resistant variety to RBSDV (Figure 1f). We also noted that the *S6RNAi* lines had no inferior effects on agronomical or developmental traits. Collectively, these results demonstrate that *S6* is an ideal target for genetic engineering of RBSDV resistance via RNAi strategy.

**Figure 1:**
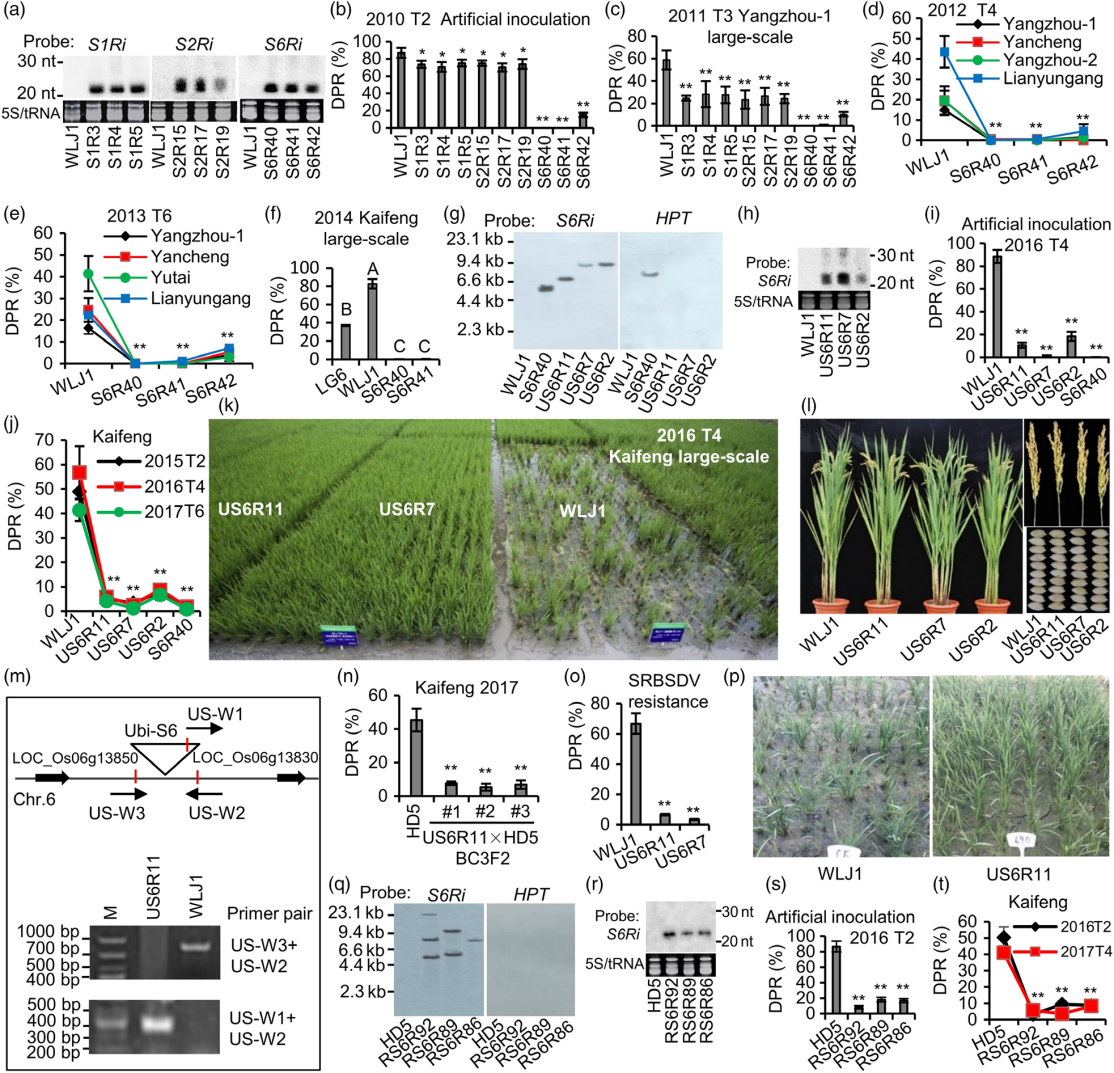
Generation of marker‐free rice with stably strong resistance to rice black‐streaked dwarf virus. (a) Accumulation of siRNAs in transgenic lines. (b‐c) Diseased/total plant ratios (DPR) of *S1RNAi*, *S2RNAi* and *S6RNAi* lines evaluated by artificial inoculation (b) and in field conditions (c). (d‐e) DPR of *S6RNAi* lines at four different locations in 2012 (d) and 2013 (e) in field conditions. (f) Comparison of RBSDV resistance between LG6 and *S6RNAi* lines. Different letters indicate significant differences according to *Duncan’s* multiple range tests (*P* < 0.05). (g‐h) Southern blots probed with *S6* and *HPT* (g) and accumulation of siRNAs (h) in *US6RNAi* T_2_ progeny. (i‐k) DPR of *US6RNAi* lines evaluated by artificial inoculation (i) and in field tests (j‐k). (l) Agronomical traits of different *US6RNAi* lines. (m) The insertion location of the *S6RNAi* construct in the genome of US6Ri11 and its verification using three specific primers, US‐W1, US‐W2 and US‐W3. (n) DPR of three introgressed homozygous lines (#1, #2 and #3) containing *US6RNAi* in the BC_3_F_2_ population from the cross of US6R11 × HD5 (recurrent parent). (o‐p) Resistance of *US6RNAi* lines to the southern rice black‐streaked dwarf virus (SRBSDV) evaluated by artificial inoculation. (q) Southern blots of *RS6RNAi* lines using *S6* and *HPT* probes. (r) Accumulation of siRNAs in *RS6RNAi* lines. (s‐t) DPR of *RS6RNAi* lines evaluated by artificial inoculation (s) and in natural fields (t). Each line was replicated three times, and each repetition contained more than 90 plants in small scale and around 1000 plants in large‐scale field tests. Disease severity was surveyed four weeks after inoculation. The RBSDV resistance level was evaluated based on the DPR which was calculated as the number of plants with typical symptoms divided by the total number of plants and multiplied by 100. 5S tRNA was used as reference for equal loading of RNA in each lane. T_2_‐T_6_ indicate different transgenic generations. *: *P* < 0.05, **: *P* < 0.01 by Student’s *t*‐test. [Colour figure can be viewed at wileyonlinelibrary.com]

Introducing the selectable marker gene in the plant transformation procedure is a wide biosafety concern in genetic engineering. To obtain marker‐free transgenic *S6RNAi* lines, we generated a double T‐DNA expression construct for *S6RNAi* driven by maize *ubiquitin‐1* promoter (*US6RNAi*) and transferred it into rice variety WLJ1. We obtained 3 independent homozygous lines (US6R11, US6R7 and US6R2) from the T_2_ plants, which were confirmed carrying single‐copy insertion and no selectable marker gene as well as the accumulation of expected siRNA by Southern and northern blot analyses (Figure 1g and h). These 3 marker‐free *US6RNAi* lines and one resistant control S6R40 were confirmed to carry high levels of RBSDV resistance in artificial inoculation test in 2016 and in multiple field trials from 2015 to 2017 (Figure 1i‐k). Notably, the 3 *US6RNAi* lines maintain the agronomic traits that are indistinguishable from those of the wild type (Figure 1l). Furthermore, through the inverse‐PCR method, we identified the insertion of the *US6RNAi* construct is located in an intergenic site at 7.69 Mp on chromosome 6 in US6R11. We further validated this insertion site by PCR using three specific PCR primers (Figure 1m; China patent: CN201911243983.0). Through backcrossing and MAS, we then transferred the *US6RNAi* construct from US6R11 into a susceptible *japonica* rice cultivar Haidao 5 (HD5, as the recurrent parent), a widely cultivated variety in Jiangsu province nowadays. We found that three introgressed lines, containing homozygous *US6RNAi* selected from BC_3_F_2_ population, all displayed markedly higher resistance to RBSDV than HD5 (Figure 1n). In addition, after the artificial inoculation of southern rice black‐streaked dwarf virus (SRBSDV), a novel species closely related to RBSDV in the genus *Fijivirus* (Zhou *et al*., 2013), we found the marker‐free *US6RNAi* lines also showed strong resistance to SRBSDV that outbroke recently in Southern China (Figure 1o and p). These results indicate that the US6R11 line has great potential for developing new resistant varieties to both RBSDV and SRBSDV.

Many experiments demonstrated that the promoter of the rice *rbcS* (*small subunit of ribulose‐1, 5‐bisphosphate carboxylase/oxygenase*) gene is useful to express target gene limited in green tissues but not in milled rice (Wang *et al*., 2015). To obtain green tissue‐expressed *S6RNAi* rice for avoiding potential effects on grains to a great extent, we transferred *S6RNAi* driven by the rice *rbcS* promoter (*RS6RNAi*) into HD5 using the double T‐DNA construct. As a result, we obtained marker‐free transgenic *RS6RNAi* lines with an ideal accumulation of the target siRNA (Figure 1q‐r). All the *RS6RNAi* rice lines displayed stable and high resistance to RBSDV (Figure 1s‐t).

In summary, our results demonstrate that RNAi targeting RBSDV *S6* conferred rice with almost full immunity to this devastating plant virus, while RNAi of *S1* or *S2* only leads to partially increased resistance. The *S6RNAi*‐mediated resistance is very stable at multiple locations throughout 8 years (2010–2017) in both artificial inoculation and field trials. Importantly, introducing the *S6RNAi* with distinct transformation vectors did not have adverse effects on agronomical or developmental traits in different rice varieties. In particular, we generated marker‐free transgenic *S6RNAi* lines in elite rice background, which should have great potential in breeding of resistant varieties to both RBSDV and SRBSDV and provide a basis for further safety evaluation and commercial application.

## Conflict of interest

The authors have declared no conflict of interest.

## Author contributions

S. Z., X. P. and G. L. W. conceived the project. Z. F., M. Y., J. Z., L. B. W., L. W., T. C., N. Z., W. X., Y. Z., Z. C. K.H. and W. L. performed the research and analysed the data. Z. F. and S. Z wrote the manuscript.
